# Supplementary material for: Curated and harmonised transcriptomics datasets of interstitial lung diseases
Source: Data Brief. 2025 Oct 14;63:112139. doi: 10.1016/j.dib.2025.112139 (PMC12581653; doi:10.1016/j.dib.2025.112139)
Supplement: Supplementary file 1 [file mmc1.zip › Supplementary_material/RNA-seq/fastQC reports/GSE184316/TRIMMED_SRR15926773_fastqc.html]

TRIMMED\_SRR15926773.fastq.gz FastQC Report 

FastQC Report

Sat 3 Dec 2022  
TRIMMED\_SRR15926773.fastq.gz

## Summary

- Basic Statistics
- Per base sequence quality
- Per sequence quality scores
- Per base sequence content
- Per sequence GC content
- Per base N content
- Sequence Length Distribution
- Sequence Duplication Levels
- Overrepresented sequences
- Adapter Content

## Basic Statistics

| Measure | Value |
| --- | --- |
| Filename | TRIMMED\_SRR15926773.fastq.gz |
| File type | Conventional base calls |
| Encoding | Sanger / Illumina 1.9 |
| Total Sequences | 11251072 |
| Sequences flagged as poor quality | 0 |
| Sequence length | 35-371 |
| %GC | 51 |

## Per base sequence quality

## Per sequence quality scores

## Per base sequence content

## Per sequence GC content

## Per base N content

## Sequence Length Distribution

## Sequence Duplication Levels

## Overrepresented sequences

| Sequence | Count | Percentage | Possible Source |
| --- | --- | --- | --- |
| AGACATCAAATCCTGCAGACAAGGGGAGCCCTCAGTCTGCAGGGCTCCAT | 86063 | 0.7649315549664957 | No Hit |
| AGGCTACACACTGAGGACTCTGTTCCTCCCCTTTCCGCCTAGGGGAAAGT | 70964 | 0.6307310094540325 | No Hit |
| GGCATCAAAAGTGATGGACTGCCCATTGCTGGAGAAGACCTTCTCTCCTA | 70767 | 0.628980065188455 | No Hit |
| TACCACTGCCACCTTCTCCATCGGCTCCACTGGCCTCGTGGTGTATGACT | 70478 | 0.6264114210628108 | No Hit |
| CAGTGGGGCCACGAGCTGAGTGCGTCCTGTCACTCCACTCCCATGTCCCT | 68314 | 0.6071776982673295 | No Hit |
| CTTCATGATGTAGCAGCAGGTGCCAGGGGCTGGCTTGTAGGCGATCAGCA | 66288 | 0.5891705252619484 | No Hit |
| GGGAAGCGGGGAATGTCTACGTGCGTATGCACGTGGCACTCTCTGCCCGA | 57049 | 0.507053905618949 | No Hit |
| CAATCCACTTGATCCCAACTCATCTCTCATTTATTTCGGCTTCTTTTATT | 53626 | 0.47663013799929466 | No Hit |
| TGATATGGTTCCTGGCAAGCCCATGTGTGTTGAGAGCTTCTCAGACTATC | 53270 | 0.4734659950625149 | No Hit |
| TTACAAAAGCAGAATAAAAGCGAAAAGAAATGAAAATGTTACACTACATT | 52887 | 0.4700618749928896 | No Hit |
| CACCTCCCCGAAGCTCAGGGAGAGCCCTGTTAGGGCCGCCTCTGGCCCTA | 52765 | 0.4689775338741055 | No Hit |
| TGATGACACCCACCGCAACTGTCTGTCTCATATCACGAACAGCAAAGCGA | 50061 | 0.4449442684217113 | No Hit |
| GGCAATGAGCGGTTCCGCTGCCCTGAGGCACTCTTCCAGCCTTCCTTCCT | 32825 | 0.29174997724661256 | No Hit |
| GTCCACGTCACACTTCATGATGGAGTTGAAGGTAGTTTCGTGGATGCCAC | 29297 | 0.26039296522144734 | No Hit |
| ACCTCTCTCTGGGCTTCTATTTCGACCGCGATGATGTGGCTCTGGAAGGC | 26754 | 0.23779067452416977 | No Hit |
| CCCTGGCTGCTACAGGAGAATAGCAGACAGGTATAGTTAAGAAACAACTT | 23926 | 0.212655291869077 | No Hit |
| ACAAGAAGTACATCAAAGATTACATGAAATCAATCAAAGGGAAACTTGAA | 22862 | 0.20319841522656684 | No Hit |
| GGCATCAAAAGTGATGGACTGCCCATTGCTAGAGAAGACCTTCTCTCCTA | 22761 | 0.20230072298888496 | No Hit |
| AGCTGCTAAGTGCTGTGTTGTCGTTCCCCCTGCTTAAAATAAAGTTGTTT | 22548 | 0.20040757005199147 | No Hit |
| ATCAAAGAAGAACATGTGATCATCCAGGCCGAGTTCTATCTGAATCCTGA | 20822 | 0.18506680963378422 | No Hit |
| CAATGGGGTACTTCAGGGTCAGGATGCCACGCTTGCTCTGGGCCTCGTCG | 20700 | 0.18398246851500016 | No Hit |
| CATATGGCTCTCCCAGAGGCAGAAGGACAATGGCTGTTTCAGGAGCTCTG | 19555 | 0.17380566047395304 | No Hit |
| CTCTCTTTCTGGCCTGGAGGCTATCCAGCGTACTCCAAAGATTCAGGTTT | 18999 | 0.16886390914572408 | No Hit |
| GTTCTGCAGCCCCTGTCATAAAAGGTTTTACTCTTTCTGGTCTCTGTTCT | 18418 | 0.16369995676856391 | No Hit |
| GGCTTGGAGCTCCTCATCTAGATGAGCTGGAAGCCCTGGAGGGCCTCTCT | 17830 | 0.15847378809770304 | No Hit |
| GGCAATGAGCGGTTCCGGTGTCCGGAGGCGCTGTTCCAGCCTTCCTTCCT | 17006 | 0.15115004152493203 | No Hit |
| AGGCGGAGAGGGTCACTTCATCTTCTACTCCTCCCTTTATGGCATTGTTG | 16775 | 0.1490969038328081 | No Hit |
| GAAACCCAGACACATAGCAATTCAGGAAATTTGACTTTCCATTCTCTGCT | 16611 | 0.1476392649518197 | No Hit |
| AATGGGCTGCCTGGAGCCCCTGGTGTCCCTGGAGAGCGTGGAGAGAAGGG | 16450 | 0.14620829019670303 | No Hit |
| GTCCACGTCACACTTCATGATGGAGTTGAAGGTGGTCTCGTGGATGCCGC | 16332 | 0.14515950124574797 | No Hit |
| ACGTTTACCTGTCCATGTCTTACTACTTTGACCGCGATGATGTGGCTTTG | 15436 | 0.1371958156520552 | No Hit |
| GCCGACAAGACCAACGTCAAGGCCGCCTGGGGTAAGGTCGGCGCGCACGC | 15353 | 0.13645810816960374 | No Hit |
| CACCAAAGTGCGAAAAGTTACTTTCAATGGACTGAACCAGATGATTGTCA | 15024 | 0.13353394236566968 | No Hit |
| CAGCATGTTCCCTCTCCTCATGAGATTGGTGAAGAAAGTATTTGGCAAAG | 14976 | 0.13310731635172185 | No Hit |
| TGCCATATCCACATGGAAAATCTCATCACCATCAAAGTCAAACATAAACT | 14505 | 0.1289210485898588 | No Hit |
| TCAGGAGACGCTCGTAGCCCTCGCGCTTCTCCTCGGCCAATTCGCGGAAG | 14356 | 0.1275967303382291 | No Hit |
| CGATCAGCACGGGCTCGTACTCTGCCACAAACTGATCACACTGCTTCTGG | 14041 | 0.12479699712169649 | No Hit |
| CGAGCCACAGCTCTGCCAGTACCCCAAGACTCAGCACTAGTCTGATGACC | 13727 | 0.12200615194712112 | No Hit |
| AGGTCTTGGTGGTGGGGAAGGACAGGAACATCCTCTCCAGGGCCTCCGCA | 13655 | 0.12136621292619938 | No Hit |
| TCAGGAGACGCTCGTAGCCCTCGCGCTTCTCCTCGGCCAGTTCGCGGAAG | 13635 | 0.12118845208705448 | No Hit |
| GTCTCAACATCTGTGTTGGGGAGAGTGGAGACAGACTGACGCGAGCAGCC | 13531 | 0.12026409572350083 | No Hit |
| CAGCAGTTTACACCTGCTAGGGTGTTCAAAGGTCAGTGCTATAGAAATTC | 13431 | 0.11937529152777619 | No Hit |
| TTCAGCCCTGATCAAGACATGAGGGAGGCAGGGGCTCAGCTGAAGAAGCT | 12750 | 0.11332253495489142 | No Hit |
| AACAAGCACAAAGCCAAGAAAACCAACGATGCCAGATACTGAATTTCTAT | 12689 | 0.11278036439549939 | No Hit |
| CTGACAGTGTATCTAGCTTTGGAAAACACAGGGGTCTGCCCTGTGAGCTG | 12391 | 0.11013172789223996 | No Hit |
| CGTGAATACCAAGACCTGCTCAATGTTAAGATGGCCCTTGACATTGAGAT | 12258 | 0.10894961831192619 | No Hit |
| GGAGAAAAACAGCACCAAGCAGGAGATCCTGGCTGCTCTTGAGAAAGGCT | 12034 | 0.106958696913503 | No Hit |
| GTTCCATATCAGAGCTGTGATCTTGAGAGCCCTCTCCTTGGCTTTCCTGC | 11669 | 0.10371456159910805 | No Hit |
| CTCCACCTACGGCGAGGGCGAGAGCGGCCCCATGGGCAACATCATGATCG | 11542 | 0.10258578027053777 | No Hit |
| CTCCGCCGCCTCCGCCGCAGACGCCGCCGCGATGCGCTACGTCGCCTCCT | 11539 | 0.10255911614466605 | No Hit |

## Adapter Content

Produced by FastQC (version 0.11.7)
